# Alteration of Protein Binding Affinities by Aqueous Two-Phase Systems Revealed by Pressure Perturbation

**DOI:** 10.1038/s41598-020-65053-6

**Published:** 2020-05-15

**Authors:** Rosario Oliva, Sudeshna Banerjee, Hasan Cinar, Christiane Ehrt, Roland Winter

**Affiliations:** 1 Physical Chemistry I - Biophysical Chemistry, Faculty of Chemistry and Chemical Biology, TU Dortmund University, Otto-Hahn-Strasse 4a, 44227 Dortmund, Germany; 2 Medicinal Chemistry – Faculty of Chemistry and Chemical Biology, TU Dortmund University, Otto-Hahn-Strasse 4a, 44227 Dortmund, Germany

**Keywords:** Molecular biophysics, Biophysical chemistry

## Abstract

Interactions between proteins and ligands, which are fundamental to many biochemical processes essential to life, are mostly studied at dilute buffer conditions. The effects of the highly crowded nature of biological cells and the effects of liquid-liquid phase separation inducing biomolecular droplet formation as a means of membrane-less compartmentalization have been largely neglected in protein binding studies. We investigated the binding of a small ligand (ANS) to one of the most multifunctional proteins, bovine serum albumin (BSA) in an aqueous two-phase system (ATPS) composed of PEG and Dextran. Also, aiming to shed more light on differences in binding mode compared to the neat buffer data, we examined the effect of high hydrostatic pressure (HHP) on the binding process. We observe a marked effect of the ATPS on the binding characteristics of BSA. Not only the binding constants change in the ATPS system, but also the integrity of binding sites is partially lost, which is most likely due to soft enthalpic interactions of the BSA with components in the dense droplet phase of the ATPS. Using pressure modulation, differences in binding sites could be unravelled by their different volumetric and hydration properties. Regarding the vital biological relevance of the study, we notice that extreme biological environments, such as HHP, can markedly affect the binding characteristics of proteins. Hence, organisms experiencing high-pressure stress in the deep sea need to finely adjust the volume changes of their biochemical reactions in cellulo.

## Introduction

One of the most common experiments in biochemistry, biophysics, medicinal chemistry, and cellular biology is testing whether a ligand binds to a protein^1–5^. Protein-ligand recognition and interaction are fundamental to many events essential to life, such as self-replication, metabolism and signal transduction. Furthermore, elucidating the nature of the forces involved in the binding processes is prerequisite for the development of new and more effective drugs in medical applications. In spite of its apparent importance, many aspects of ligand binding have not been fully explored, yet. Commonly, binding studies are carried out in dilute buffer solution and at ambient temperature and pressure. But the interior of biological cells is enriched with numerous macromolecules, such as proteins and nucleic acids, forming a highly crowded environment. Crowding affects molecular diffusion, conformation, dynamics and kinetics as well as the hydration properties of proteins^6–9^.

Further, biological cells need to orchestrate their biochemical reactions in space and time. The modulation and regulation of such processes is achieved through the compartmentalization of the cellular milieu. Besides lipid bilayer membranes, non-membrane bound compartments lacking a surrounding lipid bilayer and consisting of phase-separated liquid-like droplets have been shown to be of similar importance in recent years^10,11^. Such membrane-less droplet-like compartments, also denoted biomolecular condensates, are involved in various cellular processes, including cell growth, division, signaling, and migration^10,11^. The effects of liquid-liquid phase separation (LLPS) inducing biomolecular droplet formation on ligand binding, and biomolecular reactions in general, are still largely under-researched, however.

Here, we employed a well-characterized artificial aqueous two-phase system (ATPS) consisting of polyethylene glycol (PEG) and Dextran^12^. Due to favorable hydrophilic interactions, proteins generally partition into the Dextran enriched droplets of the ATPS. Many factors may be expected to influence the ligand binding reaction inside the Dextran-rich droplets, both favorably and unfavorably, such as conformational changes in the binding site and its dynamics as well as changes in hydration properties and the chemical activity of the ligand. Further, liquid condensates in ATPSs can locally concentrate the reaction partners, leading to an effective increase of their concentrations, or the different partitioning of protein and ligand in the coexisting phases and direct polymer-reactant interactions can alter the binding reaction.

Besides studying the effect of the ATPS system on a ligand binding reaction, we varied also the pressure of the system, for the following two major reasons:

(i) Firstly, pressure application enables modulation of intra- and inter-molecular interactions and is an important physico-chemical probe for changing the free-energy and conformational landscape of proteins, including enzymes^13–15^. Pressure favors states with a smaller partial volume. Thus, an increase of pressure will shift an equilibrium, according to Le Châtelier’s principle, towards the state that occupies the smallest possible overall volume^15^. The pressure effect on a given reversible reaction follows the relation (dln*K*/d*p*)_*T*_ = −Δ*V*/(*RT*), where *K* is the pressure-dependent equilibrium constant and ∆*V* is the associated volume change, in our case upon ligand binding. Hence, a reaction that is accompanied by a positive ∆*V* will be retarded under pressure, and conversely, a reaction enhanced by pressure will be accompanied by a negative ∆*V*. Owing to the fact that different binding sites of a protein may exhibit different structures and hydration properties, differences in their local ∆*V* values can be expected.
(ii) Secondly, pressure-axis experiments are of substantial biological and astrobiological interest. The deep oceans are populated by many organisms which are constantly exposed to pressures of more than 100 bar. Pressures of the 1000 bar level and more are reached at the deepest trenches, which are rich with various life forms as well^16,17^. Furthermore, today we know that below the surface of the Earth’s crust, entrapped in both marine sediments and terrestrial rocks, an active microbial community exists^18^. It is mandatory for such organisms to modulate the volume changes of their biochemical reactions. Henceforth, knowledge about high hydrostatic pressure (HHP) effects on biological systems is fundamental for our understanding of life in the deep sea, which is also thought to be the potential birth place of life on Earth, and for the search for life on extraterrestrial planets and moons^19^. HHP can affect a variety of biomolecular structures, including membranes, proteins, and non-canonical nucleic acid structures^13–15,20–25^. Depending on the biological system, to induce significant structural and functional changes, pressures ranging from hundreds to thousands bar must be applied. The molecular effects of pressure on binding equilibria are still largely unknown, however^26,27^.

**Figure 1:**
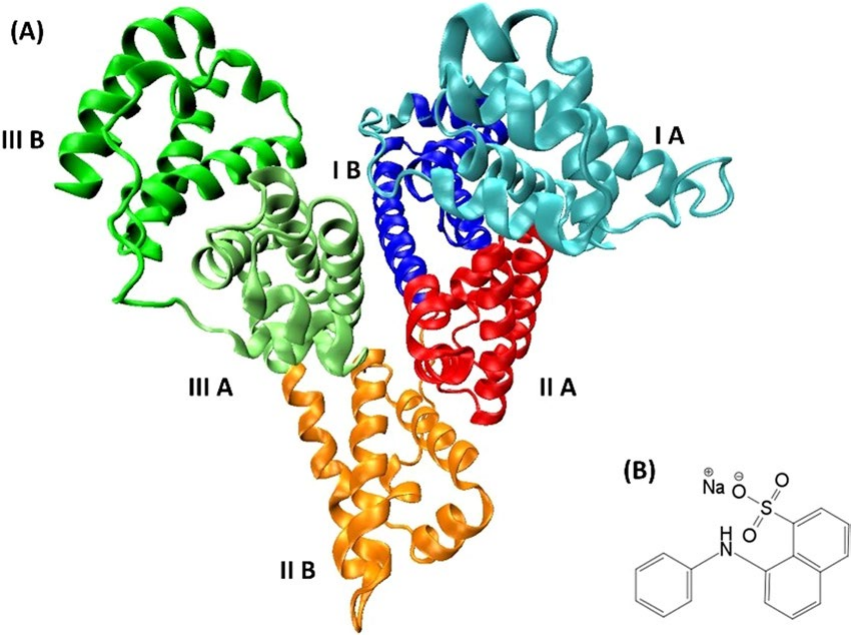
(**A**) Representation of the BSA structure (PDB code: 3V03). The six BSA subdomains are highlighted with different colors^40^. (**B**) The chemical structure of ANS.

In this work, the binding of 1-anilino-8-naphthalenesulfonate (ANS) to one of the most multifunctional proteins, bovine serum albumin (BSA, Fig. 1), was analyzed by fluorescence and circular dichroism (CD) spectroscopy, as well as by light and fluorescence microscopy. The ligand ANS is a fluorescent molecule that is widely used as probe in protein studies^28^. ANS can bind to most proteins and competes with specific natural and medically relevant ligands, such as fatty acids and ibuprofen, respectively. ANS features a shift of the maximum of emission towards lower wavelengths (blue shift), which is coupled with a strong increase of the fluorescence intensity when binding to proteins. Binding is generally attributed to the localization of ANS into proteins’ hydrophobic sites where, additionally, its dynamics is restricted (^28^ and refs. therein). However, ANS is also capable to bind to water-exposed external sites of proteins, revealing a smaller increase in fluorescence intensity compared to that observed for buried ANS molecules. It has been reported that there is an electrostatic contribution to binding at such water-exposed sites, e.g. between the sulfonate group of ANS and proximal positively charged side chains of the protein. Further, the two aromatic rings of ANS may locate in already existing or induced hydrophobic cavities of the protein^28^.

BSA-ANS binding has been previously studied at several different solution conditions, at different pH values, ionic strengths, and temperatures^29^. Studies of the combined effects of LLPS and HHP for modulating the binding constants of protein-ligand interactions such as BSA-ANS with respect to ligand binding at ambient conditions in buffer solution are still *terra incognita*. Up to now, the effect of pressure has been studied on the stability of pure LLPS systems, only^30,31^. The LLPS we are employing here, 4.6 kDa PEG and 10 kDa Dextran at 12.7 wt% and 5.5 wt%, respectively, has been shown to be pressure stable up to at least 2 kbar^32^, i.e., in the whole pressure range covered here for the ligand binding study.

## Results and Discussion

**Figure 2:**
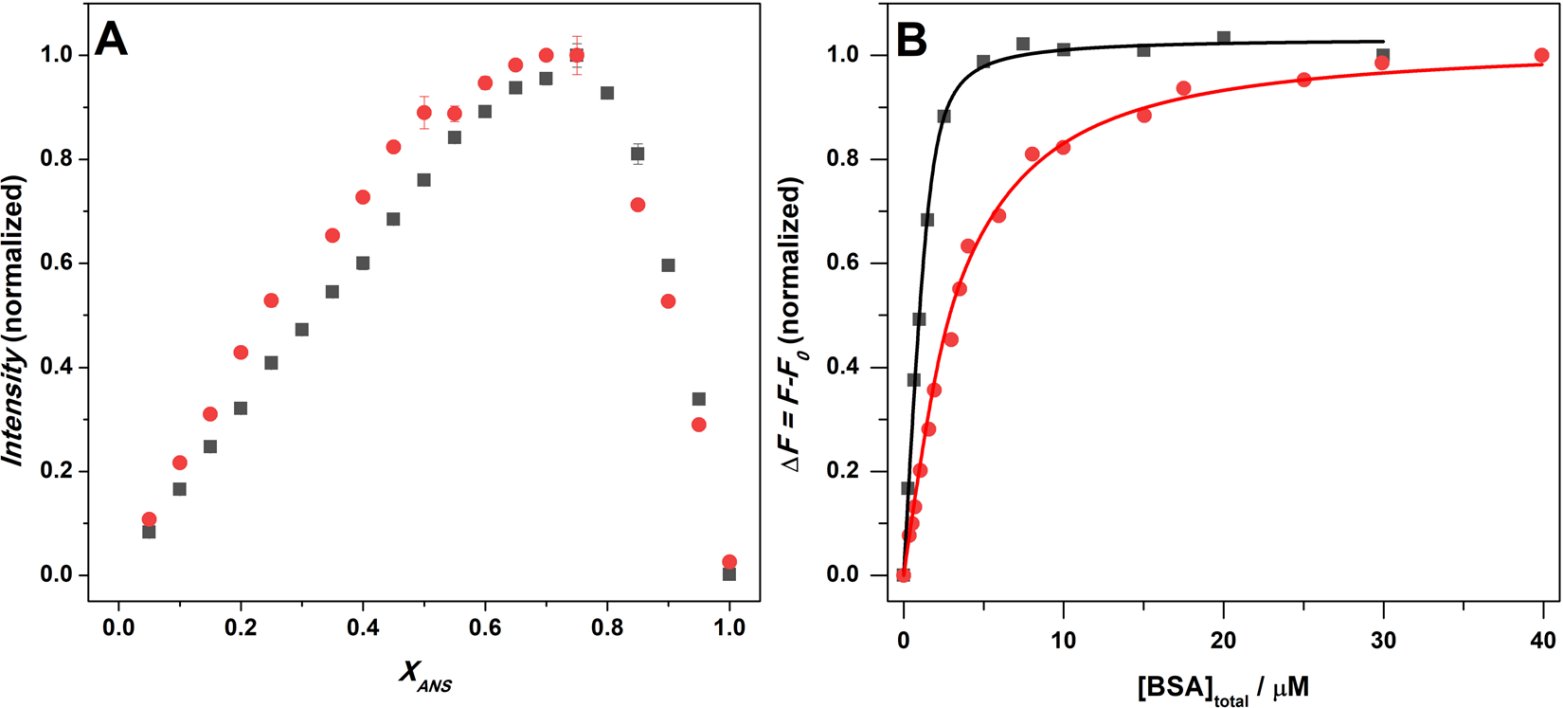
(**A**) Job’s plots for the ANS-BSA system in neat buffer (black squares) and the ATPS (red circles) obtained by means of fluorescence spectroscopy at *T* = 25 °C and *p* = 1 bar. The total concentration [ANS] + [BSA] was 35 μM. **(B**) Binding isotherms for ANS-BSA in buffer (black) and in the ATPS (red) at ambient conditions (*T* = 25 °C and *p* = 1 bar). The ANS concentration was held constant at 4.8 μM and 5.8 μM for the experiment in buffer and ATPS, respectively. The continuous lines represent best fits of the experimental points (squares and circles) according to equivalent and independent binding sites (black) and to two classes of non-equivalent and independent binding sites (red), respectively. The experiments were performed in 10 mM Tris buffer, pH 7.4. For comparison, the binding isotherms were normalized.

The strength of interaction between a protein and a ligand is quantitatively described by the equilibrium binding constant, *K*_b_. Estimation of *K*_b_ is obtained by fitting experimental data, which are correlated to the extent of protein-ligand complex formation, with the most appropriate binding model. There are different binding models available that can invoke different stoichiometries for the protein:ligand complex, from the simplest 1:1 to the 1:2 or the more complex 1:*n* stoichiometry. To obtain a good estimation of the binding constant, it is of fundamental importance to determine the stoichiometry of the protein:ligand complex. To this end, we employed the continuous variation method (also known as Job’s plot) using steady-state fluorescence spectroscopy (for details, see the Experimental Section)^33^. Here, the mole fractions of ANS and BSA were varied while keeping their total concentration constant at 35 µM. Emission spectra of ANS were recorded and a plot of the fluorescence intensity at the maximum as a function of the mole fraction of ANS was constructed. Figure 2A depicts the Job’s plots, i.e., the fluorescence intensity of ANS interacting with BSA both in buffer and in the ATPS at ambient conditions (*T* = 25 °C and *p* = 1 bar) as a function of mole fraction of ANS, *x*_ANS_.

The Job’s plot obtained in buffer is in good agreement with a previously reported one, which has been obtained at similar solution conditions^34^. The detection of one inflection point at *x*_ANS_ around 0.75 clearly demonstrates that, on average, 3 molecules of ANS are bound per molecule of BSA, pointing to a 1:3 BSA:ANS stoichiometry. In ATPS, as in neat buffer, the inflection point is centered at about 0.75, indicating that also in the ATPS, the ANS molecules are able to interact with BSA and that 3 ligand molecules are bound to one molecule of the protein on average. However, the shape of the Job’s plot in the ATPS is more intricate with respect to the one in buffer. A weak inflection point is also visible at *x*_ANS_ ≈ 0.5, pointing to a more complex binding scenario with a non-equivalence of the three binding sites in the ATPS.

Figure 2B depicts the binding isotherms for the ANS-BSA system at ambient conditions (*T* = 25 °C and *p* = 1 bar) in buffer and in the ATPS. In order to obtain binding isotherms, the fluorescence emission spectrum of ANS (at fixed concentration) in the presence of increasing BSA concentrations was recorded. The binding isotherms were obtained by plotting Δ*F* = *F* − *F*_0_ as a function of total BSA concentration (see ESI for the binding models), where *F*_0_ is the fluorescence intensity of ANS in the absence of BSA (free ANS is characterized by a low quantum yield in both media, see Fig. S1), and *F* is the fluorescence intensity recorded at each step of the titration, after mixing with BSA. The data obtained in neat buffer are well fitted with a binding model where it is assumed that all the sites are equivalent and independent. Data analysis revealed that 3.2 ± 0.2 molecules of ANS are bound to BSA with a binding constant, *K*_b_, of (4.2 ± 0.9)∙10^6^ M^−1^. The stoichiometry is the same as inferred from the Job’s plot reported above (Fig. 2A). This value is also in the range previously reported for pH ≈ 7^28,34^. According to Cattoni *et al*.^28^, the three binding sites are localized in three different hydrophobic cavities within the BSA molecule (Fig. 1A), but are not completely equivalent. Localization of ANS molecules in different BSA cavities gives rise to similar changes in their quantum yield, hence does not allow to distinguish among slightly different binding sites.

As shown in Fig. 2B, in going from buffer to ATPS, a dramatic change in the shape of the binding isotherm is observed. In the ATPS, the binding isotherm reveals a first steep increase of Δ*F* in the range 0–2.5/3 μM BSA. Then, upon further increasing the BSA concentration, the Δ*F* value increases only slightly, revealing a second binding mode. This clearly indicates that, at least, two different binding modes are operative. The experimental data are well described by a binding model where the presence of two non-equivalent and independent classes of sites is assumed (see the SI for details of the modelling). In agreement with the Job’s plot data of the ATPS (overall 1:3 BSA:ANS stoichiometry), the non-equivalent binding model predicts 2 sites for a first class and 1 site for a second class of binding events. Two ANS molecules are bound to BSA with a *K*_b1_ of (4.3 ± 2.0)∙10^6^ M^−1^ and 1 ANS molecule with a *K*_b2_ of (0.42 ± 0.21)∙10^6^ M^−1^. A comparison with the results obtained in neat buffer indicates that the binding of the first two ANS molecules does not seem to be affected by partitioning in the Dextran-rich droplet phase of the ATPS. Conversely, the binding affinity of the third ANS molecule is dramatically reduced with respect to the neat buffer data. Several reasons can be invoked to explain these results, such as different partitioning of protein and ligand in the ATPS phases, BSA self-interactions (oligomerization) or direct enthalpic interaction of crowder molecules (PEG, Dextran) with the protein, which might lead to occlusion or conformational changes of the binding site of BSA.

**Figure 3:**
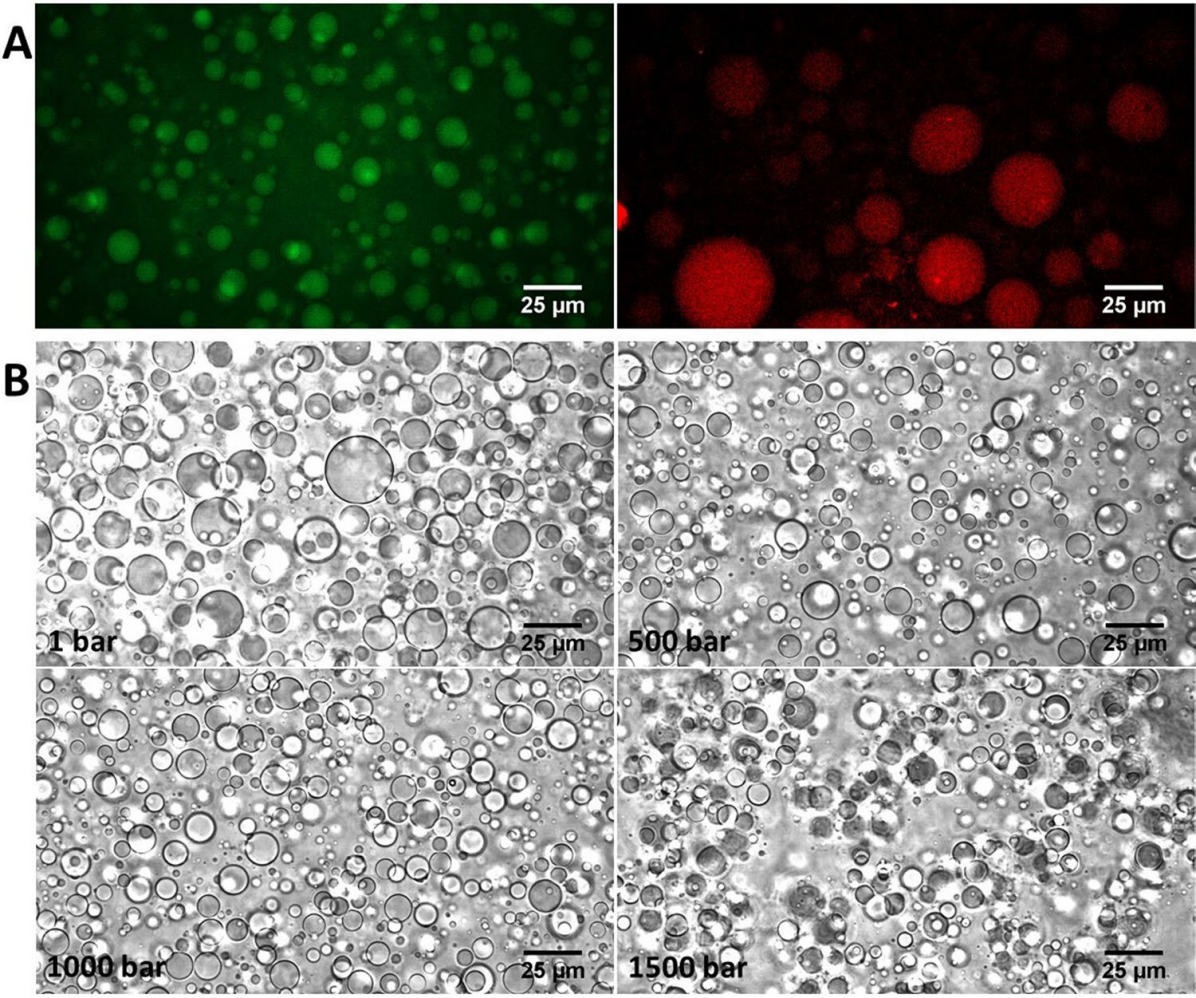
(**A**) Fluorescence microscopy pictures of FITC-BSA (left panel) and ANS (right panel) dispersed in the ATPS at ambient conditions (*T* = 25 °C and *p* = 1 bar); (**B**) Light microscopy pictures of the ATPS at selected pressures in the presence of 30 µM BSA and 5 µM ANS.

First, we verified by means of fluorescence microscopy that both BSA and ANS partition in the same phase of the ATPS. The ATPS system is composed of 4.6 kDa PEG and 10 kDa Dextran at 12.7 wt% and 5.5 wt%, respectively. The solution of these polymers is phase separated at 25 °C; one phase is enriched in Dextran, the other in PEG. According to the phase diagram^35^, the Dextran-rich phase is composed of about 30 wt% Dextran and about 3 wt% PEG, and the PEG-rich phase consists of about 14 wt% PEG and 2.5 wt% Dextran. Figure 3A shows fluorescence microscopy pictures of BSA labelled with fluorescein (FITC-BSA) and ANS in the ATPS. In a previous work, we demonstrated that the droplets in the ATPS represent the Dextran-rich phase^32^, and the ATPS is composed of droplets enriched in Dextran dispersed in a PEG-rich phase. The microscopy image in Fig. 3A shows that BSA is localized in the Dextran-rich droplets. From the picture reported in Figure 3A, we see that also ANS preferentially partitions inside the Dextran-rich phase. As control, the fluorescence intensity of ANS in the Dextran- and PEG-rich phase was evaluated. We found that they are of similar magnitude, thereby excluding the possibility that the lack of fluorescence in the PEG-rich phase is due to a low quantum yield of ANS in this phase. Hence, partitioning of the protein and the ligand in different phases cannot explain the differences detected in the binding isotherms.

It is known that BSA is able to self-oligomerize at physiological conditions and that this process is reversible^36,37^. Hence, in a crowded environment, such as that represented by the Dextran-rich phase in the ATPS, the formation of oligomers could be enhanced, which might have an impact on the interaction of the BSA with its ligands. To reveal if the ATPS could have a marked effect on the BSA self-interaction process, fluorescence anisotropy experiments were carried out. Figure S2 depicts fluorescence anisotropy data of FITC-BSA (1 µM) at increasing concentrations of unlabelled BSA in both neat buffer and the ATPS. At the initial concentration of 1 µM, the FITC-BSA is in the monomeric state at both conditions. This is supported by the observation that upon decreasing the concentration of the FITC-BSA up to 0.25 µM, the anisotropy did not change (see the inset in Fig. S2). In both media, increasing the concentration of BSA leads to an increase of anisotropy of FITC-BSA, indicating increasing protein-protein interactions, i.e., (transient) protein oligomerization. The higher anisotropy values in the ATPS compared to buffer are most likely due to an increase of the microviscosity in the ATPS system, only^38,39^. To quantitatively describe the interaction process, the experimental anisotropy data were fitted with a 1:1 binding model, corresponding to the formation of dimers (see SI for details of the modelling, with *S* being FITC-BSA and *L* the unlabelled BSA). In neat buffer, a dimerization constant of (1.8 ± 0.5)∙10^4^ M^−1^ is obtained, which is of the same order of magnitude as that determined in previous work at pH 5.8, for which a dimerization constant of ~10^5^ M^−1^ was reported^37^. In fact, considering that the pI of BSA is 5.4, at our experimental solution condition of pH 7.4, BSA should be more negatively charged, rendering the formation of dimers less favorable. In the ATPS, the dimerization constant obtained is found to be (6.3 ± 4.0)∙10^3^ M^−1^, which is, within the experimental error, slightly lower than the one obtained in neat buffer, indicating that the ATPS does not lead to a significant increase of oligomers via increasing the effective attraction of BSA molecules by an excluded volume effect enacted by the ATPS. Hence, we may assume that the dimerization/oligomerization propensity does not play a significant role in causing the differences revealed in the binding curves of BSA with ANS in neat buffer and the ATPS, respectively.

**Figure 4:**
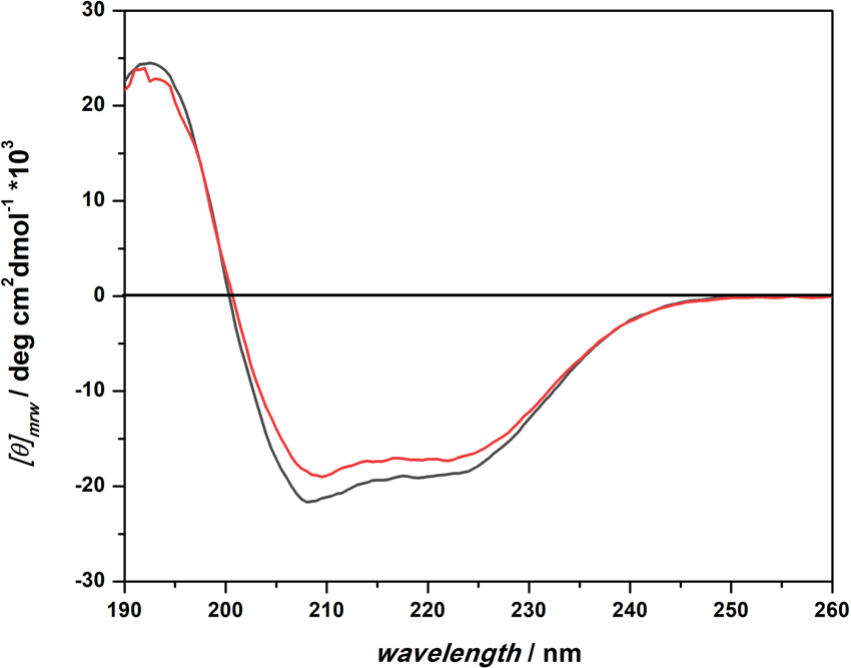
Far-UV CD spectra of 14 µM BSA in buffer (black line) and in the ATPS (red line). The experiments were carried at in 10 mM Tris, pH 7.4 at *T* = 25°C and *p* = 1 bar using a 0.01 cm path length quartz cuvette.

Finally, by using circular dichroism (CD) spectroscopy we determined the secondary structure adopted by BSA in the two different media. Figure 4 depicts far-UV CD spectra of a solution of 14 µM BSA in buffer and the ATPS. The CD spectrum of BSA in buffer reveals that the BSA adopts essentially an α-helical structure as can be inferred from the negative bands at 208 nm and 222 nm and a positive one at around 192 nm, in agreement with crystallographic data^40^. The calculation of the helix fraction yielded a value of about 59%. In the ATPS, a small change in the CD spectrum was observed. Even if the general shape of the spectrum resembles the one obtained in neat buffer solution, the intensities of the two minima are lower, indicating a small reduction of the helix content of the protein when embedded in the ATPS (~54%). The small conformational change imposed by the ATPS might be explained by an interaction of the BSA with Dextran and/or PEG within the dense droplet phase of the ATPS. In fact, it has been observed that high concentrations of Dextran can lead to a decrease of helix content of BSA, accompanied by exposure of the hydrophobic tryptophan residue to the protein surface^41,42^. Also PEG has been shown to interact with BSA, the extent of interaction depending on the PEG’s molecular weight^43,44^. It was reported that PEG is able to induce conformational changes, inducing the exposure of hydrophobic residues to the solvent^43^. At our experimental conditions, in the tightly packed Dextran-rich phase, the presence of both crowders (~30 wt% of Dextran and ~3 wt% of PEG) can be expected to lead to a similar conformational drift of the secondary structure of BSA, as revealed by CD spectroscopy. Hence, the enthalpic BSA-Dextran/PEG interaction and resulting conformational changes induced by the ATPS could lead to the changes in the binding isotherm observed. Occlusion of the region of one of the binding sites (here site number 3), might lead to both its decreasing affinity and quantum yield, and can hence explain the marked change of the binding isotherm in the ATPS compared to that obtained in buffer.

**Figure 5:**
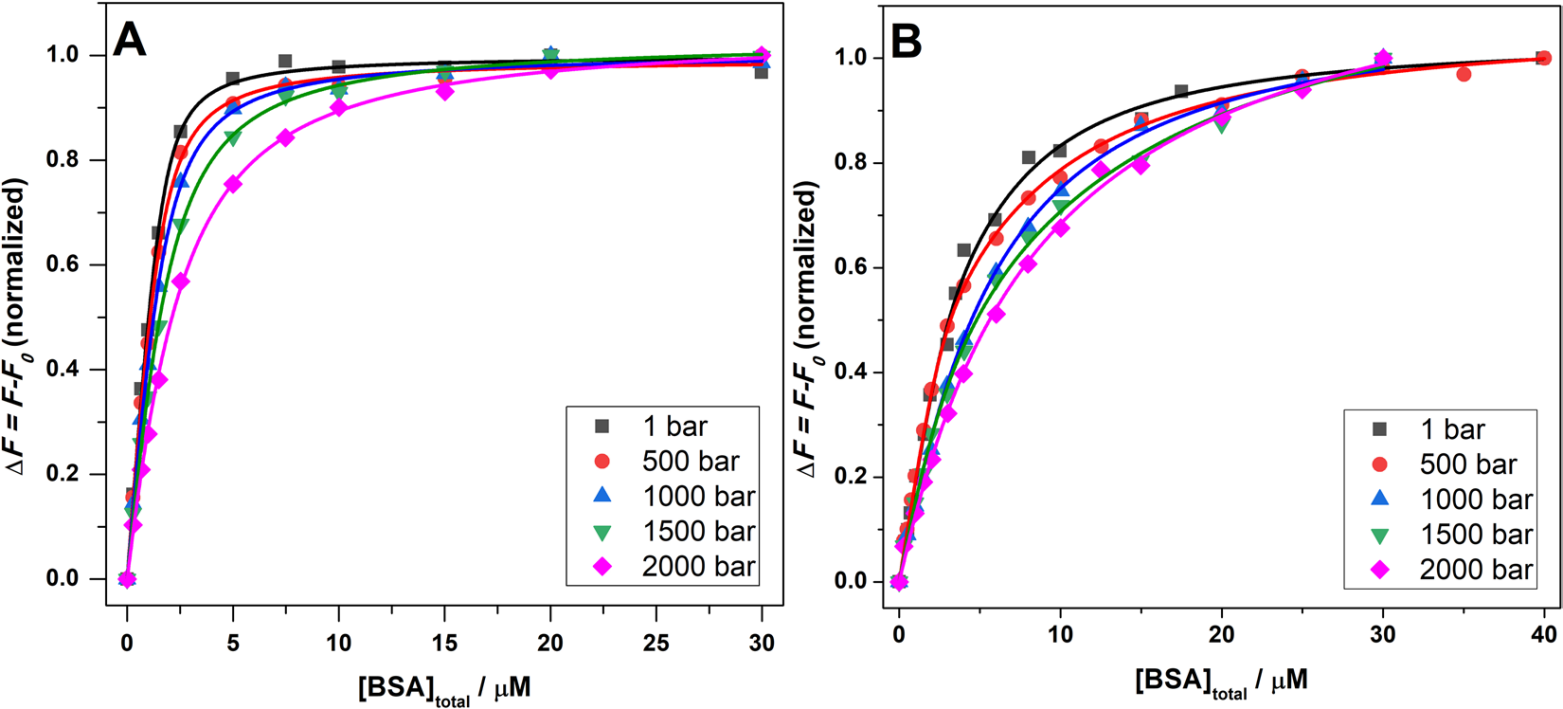
Binding isotherms for ANS-BSA in buffer (**A**) and in the ATPS (**B**) at 1, 500, 1000, 1500 and 2000 bar. The best fits of experimental data according to an equivalent and independent binding sites model (**A**) or two classes of non-equivalent and independent binding sites model (**B**) are represented by the continuous coloured lines. All the experiments were carried out in 10 mM Tris buffer, pH 7.4 at *T* = 25 °C. For comparison, the binding isotherms were normalized.

Finally, aiming to shed more light on differences in binding mode in the two solution media, we exploited the high hydrostatic pressure (HHP) effect on the binding of ANS to BSA. Generally, volume changes imposed by pressure application affect the structure, dynamics and thus also the activity of proteins. These volume changes are mainly due to variations in packing efficiency and hydration. Hence, HHP can be employed to explore the role of packing and solvation in interactions of proteins with other molecules. The experiments were carried out in both neat buffer and in the ATPS. As demonstrated in our previous work^32^, the ATPS system used here is pressure stable in the covered pressure range. Since the presence of exogenous species can influence the stability of an ATPS^45^, we verified the ATPS’s stability to HHP in the presence of BSA and ANS. As depicted in Fig. 3B, the ATPS system is stable under pressure even in the presence of BSA and ANS. Thus, a pressure effect on the BSA-ANS interaction cannot be ascribed to a change in the ATPS. Figure 5 depicts the binding isotherms obtained at pressures of 1, 500, 1000, 1500 and 2000 bar. Table 1 summarizes the binding constants obtained after data analysis. From the data reported in Table 1, it is evident that an increase of pressure leads to a decrease of the binding constant in both media, i.e., HHP disfavors ANS binding to BSA. For example, in buffer, by increasing the pressure from 1 to 2000 bar, a reduction in the *K*_b_-value by a factor of 3.5 was observed. In the ATPS, as in neat buffer, we observed a decrease of the binding constants with pressure, remarkably to different extents for the different binding sites, however. *K*_b1_ has a similar value as *K*_b_ in neat buffer at ambient pressure but shows a one order of magnitude decrease upon pressurization up to 2 kbar. Instead, *K*_b2_ is one order of magnitude smaller as *K*_b_, but shows a similar pressure dependence as the *K*_b_ in neat buffer, i.e., *K*_b2_ is reduced by a factor of 4 by increasing the pressure up to 2 kbar (see Table 1).

**Table 1:** Binding constants for the BSA-ANS interaction in buffer and the ATPS at different pressures (*T* = 25 °C).

Solvent	Buffer	ATPS	ATPS
*p*/bar	^*a*^*K*_b_/M^−1^ 10^6^	^*b*^*K*_b1_/M^−1^ 10^6^	^*c*^*K*_b2_/M^−1^ 10^6^
1	4.2 ± 0.9	4.3 ± 2.0	0.42 ± 0.22
500	3.2 ± 1.1	5.4 ± 2.5	0.23 ± 0.12
1000	2.8 ± 0.9	0.41 ± 0.20	0.22 ± 0.12
1500	1.9 ± 0.6	0.82 ± 0.40	0.090 ± 0.045
2000	1.2 ± 0.4	0.40 ± 0.21	0.099 ± 0.050

^a^The binding stoichiometry for BSA:ANS is 1:3 at each pressure; ^b^Two sites were assigned for this class with stoichiometry 1:2 BSA:ANS; ^c^One site was assigned to this class with stoichiometry 1:1 BSA:ANS.

Before addressing the volumetric properties of the BSA-ANS complexation reaction in more detail, it is mandatory to verify the conformational stability of BSA in neat buffer and the ATPS also at HHP. To this end, high-pressure FT-IR spectroscopy was carried out. Figure S3, panels a and b, shows the FT-IR spectra of BSA in buffer and in the ATPS in the amide I’ band region up to ~4000 bar. This band is sensitive to the secondary structure of the protein, rendering it useful for structural analysis. Through a deconvolution of the amide I’ band in sub-bands, it is possible to estimate the secondary structure content of the protein. The deconvolution analysis of the FT-IR spectra of BSA in buffer and the ATPS is reported in Fig. S3, panels c and d. The analysis reveals that, in neat buffer, the BSA assumes mainly an α-helix conformation (~58%), in good agreement with the CD data reported above. Further, up to 2000 bar no significant conformational changes were observed, as previously reported^46–48^. In the ATPS at ambient pressure, only minor changes were observed with respect to the neat buffer scenario, again in agreement with the CD data (Fig. 4). Increasing pressure leads to a minor conformational drift, revealing that also in the PEG/Dextran-ATPS BSA is stable under pressure and no unfolding of the protein takes place in the whole pressure range covered, leaving the binding sites intact.

**Figure 6:**
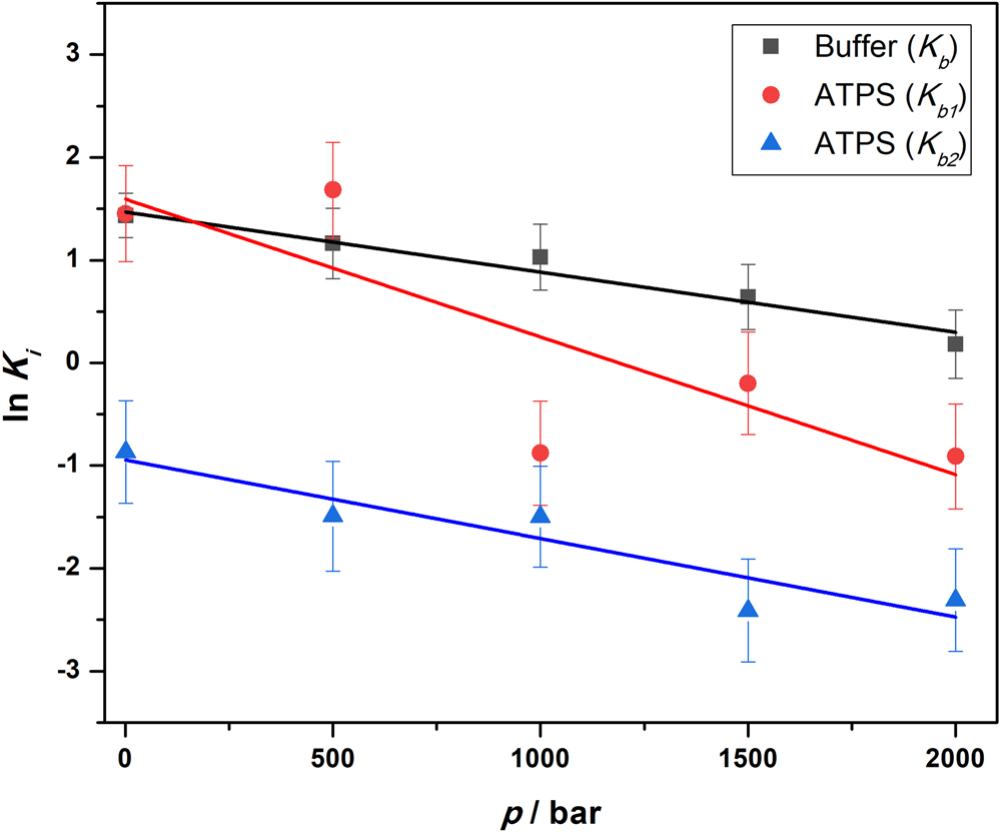
Pressure dependence of the binding constants, *K*_b*i*_, in buffer (black squares) and the ATPS (red circles for *K*_b1_ and blue triangles for *K*_b2_). From the slope of the *K*_b_(*p*) data, the binding volumes, Δ*V*_b_, can be determined. In this expanded scale, the error bars seem to be large, but the actual volume changes and the corresponding error bars are very small, in order of 1–2 water molecules for Δ*V*_b_ and a fraction of that for the error in Δ*V*_b_, only.

To shed more light on the changes imposed by pressure on the ligand binding in buffer and in the ATPS, we carried out a volumetric analysis of the results obtained. According to (dln*K*_b_/d*p*)_*T*_ = −Δ*V*_b_/(*RT*), where *K*_b_ is equilibrium binding constant, *R* the gas constant, and Δ*V*_b_ the volume change upon ligand binding, it is possible to determine the volume change involved in BSA-ANS complex formation from the pressure dependence of the equilibrium binding constant^15,32^. The binding volume (per mole of site), Δ*V*_b_, refers to the difference between the volumes of the protein–ligand (*SL*) complex and the unbound state (*S* + *L*). Figure 6 depicts the pressure dependence of the binding constants, from which the site-specific Δ*V*_b_-values have been determined.

The Δ*V*_b_-value obtained for the three binding sites of ANS in neat buffer solution is 14.5 ± 1.5 mL mol^−1^ (please recall that the molar volume of one water molecule is just 18 mL mol^−1^), i.e., the volume change is very small. The positive value of Δ*V*_b_ indicates that the volume of the complexed state is larger than that of ANS and BSA, leading to the observed decrease of binding constant at high pressures (Table 1). The positive binding volume is most likely due to an increase in void volume when ANS is bound in the binding pockets of BSA, and/or due to the release of bulk water upon partial desolvation of the interacting partners upon binding (please note that the density of bulk water is smaller than that of bound hydration water^15,47^). In the ATPS, the binding volumes are 33.2 ± 12.5 mL mol^−1^ and 18.9 ± 4.1 mL mol^−1^ for the first (*K*_b1_, *n* = 2) and for the second (*K*_b2_*, n* = 1) class of binding sites, respectively, i.e., the sites differ not only in the magnitude of the binding constant but also in packing (geometry) and/or solvation properties, which determine the magnitude of the Δ*V*_b_-values.

**Figure 7:**
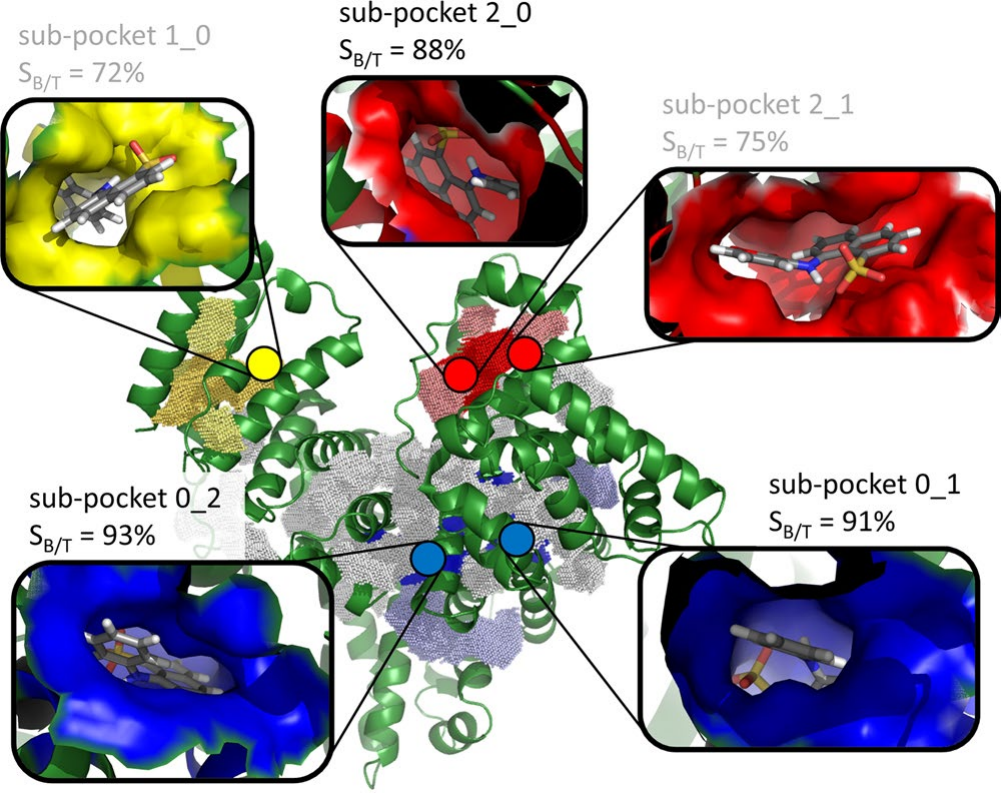
Visualisation of the detected pockets and the most probable ANS binding sites. All detected pockets are depicted as dotted grids. The three visually inspected sites which led to the highest scores after consensus docking are highlighted in blue (pocket 0), yellow (pocket 1), and red (pocket 2). The sub-pockets with the highest ranks according to the scores are highlighted in bright colors (sub-pockets 0_1, 0_2, 2_0, 2_1, and 1_0). The predicted ANS binding mode for these sub-pockets and a representation of the pocket surface are given in the inserts to highlight the exposed nature of sub-pockets 1_0 and 2_1. Additionally, the ratio of the buried and total ligand surface (*S*_B/T_) is given as calculated with PLATINUM^50^.

Finally, we performed a combined binding site detection and docking approach to reveal potential ANS binding sites of BSA and to tentatively identify the possible ANS binding site which could be affected by the ATPS. Overall, DoGSite^49^ detected 36 sub-pockets on the surface of the protein structure with the PDB-ID 4f5s (Fig. 7 and Table S1). Using those pockets for a consensus docking and scoring approach with ANS led to the identification of five promising binding sites. They were visually inspected to evaluate their potential to accommodate ANS (Fig. 7). Additionally, the protein-ligand interaction descriptors were calculated using PLATINUM^50^. The pocket with the highest rank was unique and highly buried, hinting towards an appropriate site (sub-pocket 2_0), located in the subdomain IB. In contrast, the ligand ANS was highly solvent-exposed in the second-highest ranked binding pocket (sub-pocket 2_1). The following two buried pockets (sub-pockets 0_1 and 0_2 in the subdomain IIA) were also proposed as potential binding sites^28^. They are characterized by a high buriedness and hydrophobicity. Notably, the fifth-highest ranked binding site (sub-pocket 1_0, in the subdomain IIIA) was also predicted in a study by Cattoni and co-workers^28^. However, the former analysis was based on a homology model of the protein and the predicted ANS binding mode in the present study is characterized by a high solvent exposure in the crystal structure. The remaining pockets led to low docking scores and/or were highly solvent-exposed. Consequently, we propose that the three potential buried ANS binding sites detected in our fluorescence experiments are sub-pockets 2_0, 0_1 and 0_2. An inspection of Fig. 7 reveals that the sub-pockets 0_1 and 0_2 are close to each other and located in the subdomain IIIA, which is well buried in the centre of the protein structure (for these two sites, the ratio of the buried and total ligand surface, *S*_B/T_, is higher than 90%). Most likely, these are the two sites with the same binding affinity (*K*_b1_) detected in our fluorescence experiment. Instead, the sub-pocket 2_0 in the subdomain IB is localized in the more solvent exposed subdomain IB. This site possesses a higher solvent accessibility compared to the other two sites. Since we observed small conformational changes of BSA in the presence of the ATPS, it is reasonable to suggest that the ATPS polymers induce, through direct soft enthalpic interactions, some changes in the more exposed IB subdomain, leading to a change in its quantum yield and revealing a different, second binding mode as suggested by the experimental data.

## Summary and Conclusions

In conclusion, we observe a marked effect of the ATPS on the binding characteristics of BSA as a prolific binder and transporter of small molecules such as ANS. Not only the binding constants change in the ATPS system, but also the integrity of the three binding sites (Fig. 7) is lost, which is most likely due to soft enthalpic interactions of the BSA with the Dextran/PEG crowder in the dense droplet phase of the ATPS. The binding characteristics changes upon surface sticking to ATPS components, leading to a non-equivalence of binding sites. It is very likely that this effect of the ATPS plays an important role in many other cellular liquid-phase droplets serving as membrane-less compartments in biological cells^10,11,45^. In more general terms, interaction of the protein with the ATPS can not only result in changes in the dynamical properties of the protein owing to the strong confinement imposed by the ATPS components^51^, but also to a shift of the population of different conformational substates of the funnel-like free-energy landscape of the protein. This conformational shift might lead to a change in the height of the free energy barrier of binding or even to a change in specificity.

Using pressure modulation, differences in binding sites can be unravelled by their different volumetric and hydration properties. Most likely generally, an increase of pressure leads to a decrease of the binding affinity, which is due to an increase of the partial volume of the binding complex with respect to the partial volumes of the binding partners. The increase of binding volume, Δ*V*_b_, is due to imperfect packing of the binding complex and to changes in hydration properties upon ligand binding. In further studies it remains to be seen if the binding volumes determined by this method can be correlated to the strength of ligand binding, which might have interesting ramifications in the search for effective protein drugs. As the enthalpy-entropy compensation does not lead to dramatic changes in the binding free energy of ligands, Δ*G*_b_, the discrimination between the entropic and enthalpic contributions to Δ*G*_b_ is difficult in rational drug design^4^. Knowledge of complementary Δ*V*_b_ data might be helpful in that respect.

Finally, regarding the vital biological relevance of these pressure studies, we notice that extreme biological environments, such as HHP, can markedly affect the binding characteristics of proteins, leading not only to changes in binding affinity (generally probably mainly decreasing the *K*_b_-value), but also to changes in the nature of the binding sites. Unlike their terrestrial relatives, organisms thriving in the deep sea at multi-hundred bar pressure levels need to adjust the volume changes accompanying their biochemical processes. Hence, pressure-axis experiments on biochemical processes in biological LLPS systems are mandatory to comprehend how organisms are coping with such pressure stress.

## Experimental Procedures

### Materials

Lyophilized powder of the protein bovine serum albumin (BSA), the fluorophore 8-anilinonaphthalene-1-sulfonic acid (ANS), polyethylene glycol (PEG) with a molecular weight (MW) of 4.6 kDa and fluorescein-labelled BSA (FITC-BSA) were obtained from Sigma Aldrich Chemical, Germany. Dextran 10 kDa was purchased from Carl Roth, Germany. All the chemicals were used without any further purification. Solutions were prepared in pressure-stable Tris buffer (10 mM Tris-HCl, pH 7.4) and deionized water was used for the buffer and the sample preparations.

### Sample preparation

A stock solution of bovine serum albumin (BSA, MW of 66 kDa, 583 residues) was prepared by dissolving the protein in Tris buffer. The exact concentration of the solution was determined by measuring the absorbance at 280 nm by means of UV/Vis spectroscopy (UV-1800 spectrometer from Shimadzu Corporation) and using a molar extinction coefficient of 43600 M^−1^ cm^−1^. The stock solution of the fluorophore ANS was prepared by dissolving it in water. Subsequent dilution in buffer was performed to determine the exact concentration. The concentration was determined by measuring the absorbance using a molar extinction coefficient of *ε*(350 nm) = 4950 M^−1^ cm^−1^. The ATPS, composed of PEG 4.6 kDa and Dextran 10 kDa, was prepared by mixing appropriate amounts of PEG and Dextran solutions dissolved in buffer. At these conditions, coexisting Dextran-rich and PEG-rich phases were obtained at room temperature. The final concentration of PEG and Dextran were 12.7 wt% and 5.5 wt%, respectively. For Fourier-transform infrared (FT-IR) spectroscopy, all measurements were performed in D_2_O as solvent owing to the overlap of the H_2_O deformation vibration with the amide I’ band. To this end, Tris-D_2_O or Tris/ATPS-D_2_O buffer with pD = 7.4 was used. The protein solutions were prepared by adding 6 mg BSA to 100 µL D_2_O buffer. Subsequently, the samples were heated to 45 °C for 10 min and lyophilized. After lyophilization, the samples were dissolved again in 100 µL Tris-D_2_O or Tris/ATPS-D_2_O buffer with pD = 7.4, resulting in a protein concentration of ∼6 wt%.

### Fluorescence spectroscopy – binding assay

The interaction between BSA and ANS was followed by means of steady-state fluorescence spectroscopy using a K2 fluorometer from ISS, Inc. (Champaign, IL, USA) at the temperature of 25 °C. The instrument is equipped with a xenon arc lamp as light source. The titrations were performed by recording the ANS emission spectra by exciting the solutions at 350 nm and recording the emission intensities in the range 400–550 nm. The width slits of the excitation and emission monochromators were both set to 8 nm. Briefly, a series of solution with a fixed concentration of ANS at ~ 5 µM were prepared and the concentration of BSA was varied between 0 and ~40 µM. Then, using a plot of Δ*F* = *F* − *F*_0_ (where *F* is the fluorescence intensity of ANS at the maximum in the presence of BSA and *F*_0_ is the intensity of ANS in the absence of BSA) as a function of total BSA concentration and fitting the experimental data according to the binding models described below, we obtain an estimation of the binding constants. For the pressure dependent measurements, the high-pressure cell system from ISS and quartz cuvettes were used. The pressure was controlled by means of a manual pump and water was used as pressurizing fluid. A pressure range from 1 bar to 2000 bar was explored. The ANS and BSA solutions were mixed, vortexed and then filled into the sample cell, which was sealed with DuraSeal^TM^ laboratory stretch film and placed into the high-pressure vessel.

### Fluorescence spectroscopy – job’s plot

The Job’s method, also known as the method of continuous variation, is a very useful method for the evaluation of binding stoichiometries in complexes^33,52^. In this method, the mole fractions of both the species are varied while keeping their total concentration constant. In this case, the total concentration of ANS and BSA was kept constant at 35 μM and the mole fraction of ANS (*x*_ANS_) was varied from 1 to 0.05. The total volume of the solution was 100 μL. The fluorescence intensity of the BSA-ANS solutions was measured by setting the excitation wavelength at 350 nm and recording the emission in the range 400–550 nm by using a 0.3 cm path length quartz cuvette. Then, the intensity at the maximum was plotted versus the ANS molar fraction to obtain an estimation of the binding stoichiometry. The experiments were performed in neat buffer and ATPS at ambient conditions (*T* = 25 °C and *p* = 1 bar), by using the same instrumentation as described above.

### Fluorescence spectroscopy – anisotropy

Fluorescence anisotropy measurements were carried out to verify the propensity of BSA to self-interact in both solvents, neat buffer, and ATPS. Briefly, a 1 µM FITC-BSA solution was mixed with ~60 µM of unlabelled BSA for the sample in buffer, and with ~50 µM for the ATPS sample. Then, a successive dilution of the samples was performed to reach the desired BSA concentration. At the same time, a small amount of FITC-BSA was added to keep its concentration constant at 1 µM. The experiments were performed by exciting the sample at 480 nm and recording the fluorescence anisotropy at 560 nm using a 0.3 cm path length quartz cuvette at ambient conditions.

### Circular dichroism spectroscopy

Far-UV circular dichroism spectra were acquired on a Jasco J-715 (Jasco Corportation, Tokyo, Japan). The spectra of a 14 µM BSA solution in buffer and ATPS were recorded using a 0.01 cm path length quartz cuvette at the temperature of 25 °C in the range 260–190 nm for the buffer sample. Due to light scattering of the ATPS, the spectrum was acquired up to 195 nm, only. For each sample, a background blank (buffer or ATPS) was subtracted. The recorded spectra are the results of 3 accumulations and they were normalized per mole of residue. The estimation of the protein helix fraction (*f*_H_) was performed by evaluating the mean residue ellipticity at 222 nm as described elsewhere^53^.

### Light and fluorescence microscopy

Light and fluorescence microscopy experiments were carried out using an Eclipse TE2000-U (Nikon Inc.) optical microscope with a Nikon CFI Plan Apo Lambda 10x objective coupled to an TIS DMK 23UX249 camera. For fluorescence microscopy recordings, FITC-BSA and ANS were used. For the pressure-dependent light microscopy studies, a home-built high-pressure microscope was used^31^. The pressure was generated hydrostatically by a high-pressure hand pump with water as pressure-transmitting fluid. Flat diamond windows were used as optical window material on both sides.

### Fourier-transform infrared (FT-IR) spectroscopy

The pressure-dependent FT-IR measurements were carried out using a Nicolet 6700 FTIR spectrometer (Thermo Fisher Scientific, Waltham, MA, USA) equipped with a nitrogen-cooled MCT detector. The measurements were done using a diamond anvil cell with type IIa-diamonds. As pressure indicator, BaSO_4_ was used, which has a characteristic pressure sensitive sulphate band at 983 cm^−1^. For all measurements, the spectral resolution used was 2 cm^−1^. The apodization was performed by a Happ-Genzel function. The data obtained were processed with the GRAMS/AI 8 software (Thermo Fisher Scientific, Waltham, MA, USA). The respective buffer spectrum was subtracted from the sample spectrum. After baseline correction, the amide I’ band was normalized. In the first step of the secondary structure analysis, the maxima of the sub-bands were determined. To this end, Fourier-self deconvoluted spectra were compared with second derivative of the spectra. Then, the number, position and half-width of the sub-bands were determined. The fitting of the sub-bands to the amide I’ band was completed using mixed Gaussian-Lorentz functions. The variation of each sub-band position was limited to ± 2 cm^−1^, and the area of each sub-band corresponds to the percentage of the respective secondary structure of the protein.

### Docking studies

The structure of BSA with the PDB-ID 4f5s, which has the highest resolution of all publicly available BSA structures, was used for the analyses described herein. All molecules but the protein were removed. Hydrogen atoms were added to the protein structure using the “Protonate3D” method in MOE2019 (Molecular Operating Environment (MOE). 1010 Sherbooke St. West, Suite #910, Montreal, QC, Canada, H3A 2R7: Chemical Computing Group ULC; 2019.) and the structure was saved as PDB file. This file was uploaded to the ProteinsPlus server^54^ and subjected to a DoGSite pocket identification^49^. This led to the assignment of 17 pockets. However, some of the detected pockets were large (the largest pocket had a volume of 1876.74 A³) and not suited for the subsequent site-based docking. Consequently, a sub-pocket detection with DoGSite was performed. The 36 resulting sub-pockets were used for molecular docking studies with GOLD^55^. To this end, the ligand ANS was subjected to hydrogen atom assignment and energy minimisation (MMFF94x force field) using MOE2019. The molecular docking with GOLD was performed with default settings applying the following changes: search efficiency of 200%, 200 genetic algorithm runs, disabled option for early termination. The binding sites were defined based on the DoGSite-predicted sub-pockets. The residue names and numbers of the DoGSite-derived residue-defined sub-pockets were extracted and written to GOLD-compatible residue list files. Four docking runs with ANS into all identified sub-pockets were performed applying the scoring functions Chemscore, ChemPLP, ASP, and Goldscore. The scores of the best-scored pose per scoring function and pocket were extracted and used as the ranking measure. Pockets with the highest mean ranks for all four scoring functions were finally visually inspected to identify the most probable BSA binding sites for ANS. Protein-ligand interaction descriptors were calculated with the PLATINUM webserver^50^.

## Supplementary information

Supplementary Information.
