# Supplementary material for: Alteration of Protein Binding Affinities by Aqueous Two-Phase Systems Revealed by Pressure Perturbation
Source: Sci Rep. 2020 May 15;10:8074. doi: 10.1038/s41598-020-65053-6 (PMC7228918; doi:10.1038/s41598-020-65053-6)
Supplement: Supplementary file 1 — Supplementary Information. [file 41598_2020_65053_MOESM1_ESM.docx]

**Alteration of Protein Binding Affinities by Aqueous Two-Phase Systems Revealed by Pressure Perturbation**

Rosario Oliva,^#^* Sudeshna Banerjee,^#^ Hasan Cinar,^#^ Christiane Ehrt^+^, and Roland Winter^#^*

^#^ Physical Chemistry I - Biophysical Chemistry, Faculty of Chemistry and Chemical Biology,

TU Dortmund University, Otto-Hahn-Strasse 4a, 44227 Dortmund, Germany

^+^ Medicinal Chemistry, Faculty of Chemistry and Chemical Biology, TU Dortmund University, Otto-Hahn-Strasse 4a, 44227 Dortmund, Germany

*E-mails: rosario.oliva@tu-dortmund.de; roland.winter@tu-dortmund.de

**Supplementary Information**

**Binding Models**

**Equivalent and Independent Binding Sites**

In the following treatment, *S* indicates the substrate, whose concentration is held constant during the titration with the ligand, *L*. In case that the interaction process can be described by an equivalent and independent sites mode, i.e., there are *n* equivalent and independent sites on the substrate *S* where *L* can be bound*,* the reaction where *n* ligands *L* are bound to one *S* molecule, can be written as:

$$S+nL \to{SL}_{n}$$

For this model, it is possible to define a microscopic binding constant, *K*_b_, representing the binding constant per site (i.e., *K*_b_ expresses the equilibrium that would be measured if only one ligand can be bound), as^1^:

$$K_{b}=\frac{[SL_{1}\ldots L_{n}]}{\left[ S \right]\left[ L_{1} \right]\ldots[L_{n}]} (Eq. 1)$$

Here, [*SL*_1_*…L_n_*] represents the concentration of the complex which is formed through the occupancy of the single sites on the substrate, [*S*] is the free substrate concentration, and [*L*_1_]…[*L_n_*] is the product of concentrations of free ligands. At each step of the titration, once the equilibrium has been reached, according to the law of conservation of mass, *K*_b_ can be written as:

$$K_{b}=\frac{[x]}{\left( {[S}_{0}]-[x] \right)\left( [L_{0}]-n[x] \right)} (Eq. 2)$$

where [*S*_0_] and [*L*_0_] denote the total concentrations of the substrate and of the ligand, respectively, [*x*] is the unknown concentration of the complex formed ([*SL*_1_*…L_n_*]), and *n* indicates the number of moles of *L* bound per mole of *S*. After rearrangement of the equation, one arrives at the following quadratic expression:

$$ax^{2}-bx+c=0 (Eq. 3)$$

where: $a=nK_{b}; b=\left( 1+n{[S}_{0}]K_{b}+{[L}_{0}]K_{b} \right); c={[S}_{0}][L_{0}]K_{b}$.

Solution of Eq. 3 allows to calculate the concentration of the complex ([*SL*_1_*…L_n_*]) formed at each step of the titration:

$$x=-\frac{\sqrt{b^{2}-4ac}-b}{2a} (Eq. 4)$$

The degree of binding, also denoted the fractional saturation, *θ*, which describes the fraction of bound substrate, is given by:

$$\theta=\frac{\left[ SL_{1}\ldots L_{n} \right]}{{[S}_{0}]} (Eq. 5)$$

which varies from 0 (no *L* bound to *S*) to 1 (all the sites on *S* are occupied by *L*).

From Eqs. 4 and 5, where *x =* [*SL*_1_*…L_n_*], we obtain the final equation which describes the ligand binding process:

$$\theta=\frac{1}{{[S}_{0}]}\left( -\frac{\sqrt{b^{2}-4ac}-b}{2a} \right) (Eq. 6)$$

The fractional saturation is directly linked to the changes in a measured physical variable, Δ*Y*, through:^2^

$$\theta=\frac{\Delta Y}{(\Delta Y)_{\mathrm{total}}} . (Eq. 7)$$

Here, Δ*Y* can be a change in any physical observable, such as UV/Vis absorption or fluorescence intensity. It is important to note that Eq. 7 is valid only if the change in *Y* is linear with the extent of binding, i.e., the formation of the complex. Here, it is assumed that only the substrate *S* gives rise to a change in the observable *Y* when *L* is added to the solution. With this assumption in mind, Δ*Y=Y-Y*_0_ and (Δ*Y*)_total_*=Y*_sat_*_._-Y*_0_, where *Y* is the value of the physical observable at each step of the titration, *Y*_0_ is its value in the absence of *L* and *Y*_sat_*_._* is the value of *Y* at the end of the titration, when *S* is fully saturated with *L*. By combining Eqs. 6 and 7, we will obtain the final binding isotherm, assuming that *n* ligands *L* can be bound to *S* independently of each other and that all the sites are equivalent, i.e., can be described by the same binding constant:

$$\frac{\Delta Y}{(\Delta Y)_{\mathrm{total}}}=\frac{1}{{[S}_{0}]}\left( -\frac{\sqrt{b^{2}-4ac}-b}{2a} \right) . (Eq. 8)$$

To apply Eq. 8, it is necessary to reach the saturation level. Typically, the experiment is performed by recording a change in *Y* of the substrate after successive addition of the ligand and the binding isotherm resembles a rectangular hyperbola. At the beginning of the titration, when [*S*_0_] *>* [*L*_0_]*,* a linear variation of *Y* is observed. Then, when the concentrations are comparable, the changes in *Y* are less pronounced and the linearity is lost. Finally, when [*S*_0_] *<* [*L*_0_], a plateau is reached. In this region, successive additions of the ligand do not cause a change in *Y*, meaning that the saturation has been reached. Sometimes, it is not possible to reach the saturation, and consequently, *θ* cannot be calculated directly. This could happen in the case of weak binding. In this case, (Δ*Y*)_total_ is a fit parameter as well.

**Two Classes of Non-equivalent and Independent Binding Sites**

The binding Eq. 8 can be easily extended in case that two classes of sites are present. In this model, there are *n*_1_ and *n*_2_ sites of the first and the second class, respectively. Each class is described by a microscopic binding constant, *K*_b1_ and *K*_b2_, respectively. Since in this model it is assumed that the two classes of sites are fully independent, i.e., the binding of a ligand of the first class has no influence on the binding of a ligand of the second class, and *vice versa,* the final result for the number of total sites is^2^:

$$\frac{\Delta Y}{(\Delta Y)_{\mathrm{total}}}=\frac{\left\{ \frac{1}{{[S}_{0}]}\left[ \left( -\frac{\sqrt{b^{2}-4ac}-b}{2a} \right)+\left( -\frac{\sqrt{g^{2}-4fh}-g}{2f} \right) \right] \right\}}{(n_{1}+n_{2})} (Eq. 9)$$

where:

$a=n_{1}K_{b1};$ $b=\left( 1+n_{1}{[S}_{0}]K_{b1}+[L_{0}]K_{b1} \right);c={[S}_{0}][L_{0}]K_{b1}$;

$f=n_{2}K_{b2};$ $g=\left( 1+n_{2}{[S}_{0}]K_{b2}+{[L}_{0}]K_{b2} \right); h={[S}_{0}][L_{0}]K_{b2}$;

If the saturation cannot be reached, $(\Delta Y)_{\mathrm{total}}$is an additional fit parameter. The above equations (Eqs. 8, 9) can be easily implemented in commercially available software, such as Microsoft Excel® or Origin from OriginLab®.

**Additional Figures and Tables**


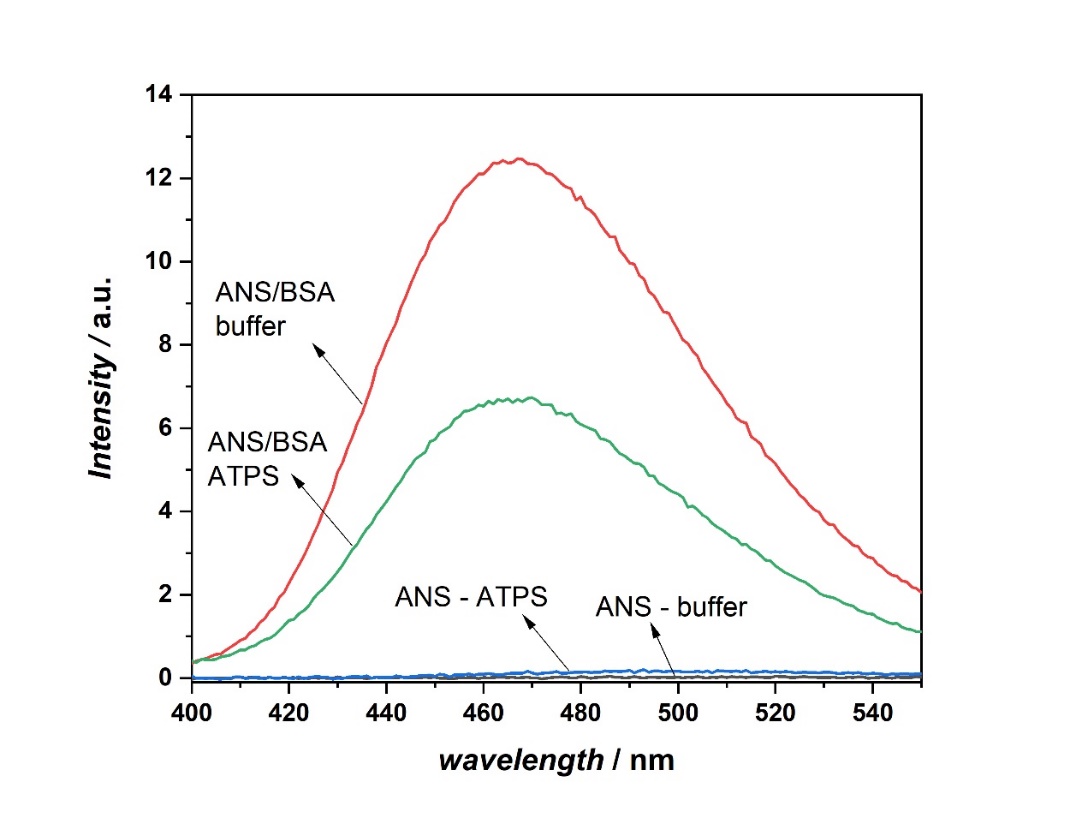


**Figure S1** Fluorescence emission spectra of ANS (4.6 µM) in the absence and in the presence of 20 µM BSA in neat buffer and in the ATPS composed of PEG and Dextran. The fluorescence of ANS in the absence of BSA is characterized by a low quantum yield in both media.


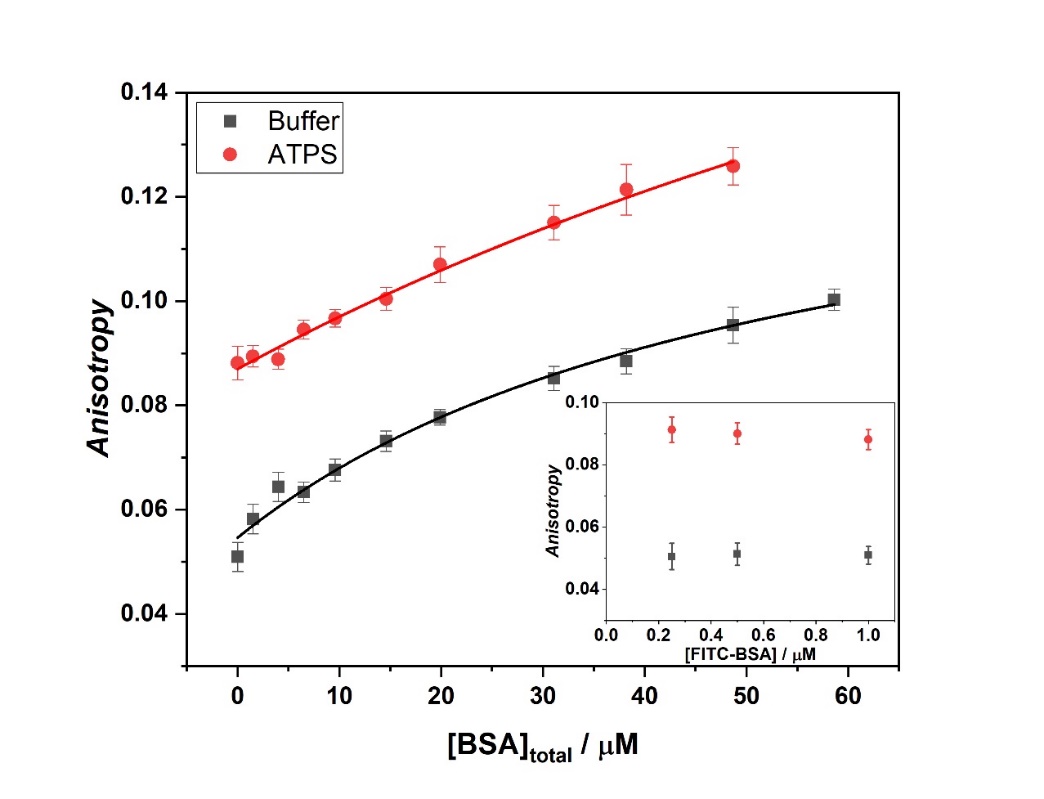


**Figure S2** Fluorescence anisotropy of 1 µM FITC-BSA at increasing concentrations of unlabelled BSA in buffer (black squares) and in the ATPS (red circles) at 25 °C and 1 bar. The lines represent the best fit of the experimental points according to a 1:1 binding model which describes, in this case, the dimer formation. The inset shows the anisotropy values of FITC-BSA samples at 0.25, 0.5 and 1 µM in buffer (black squares) and in the ATPS (red circles).

^
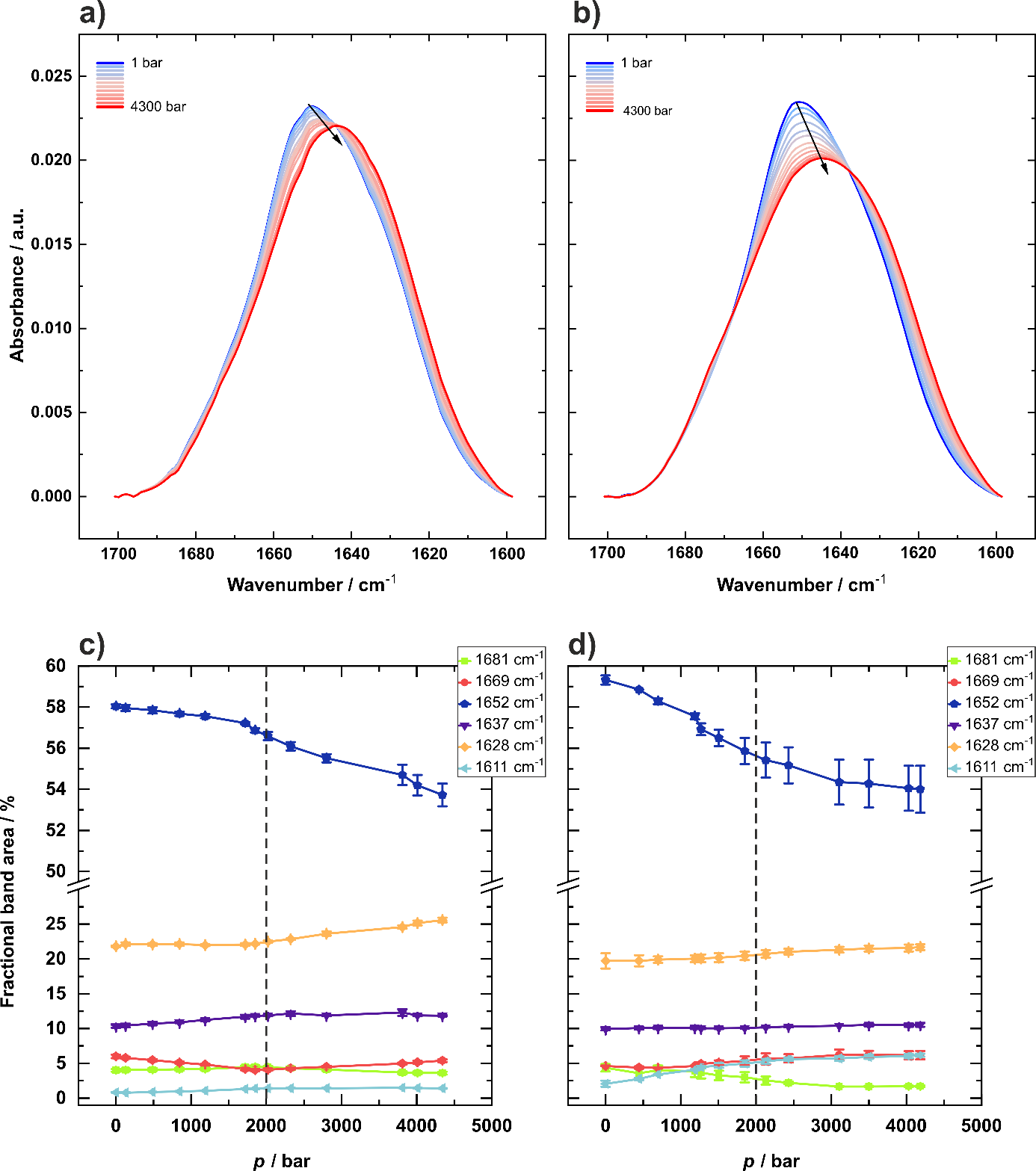
^

**Figure S3** Pressure-dependence of the FT-IR spectra of a) BSA and b) BSA-ATPS in D_2_O buffer at *T* = 25 °C in the pressure range from 1 bar to 4.3 kbar. Pressure dependence of the fractional band areas of secondary structure elements of c) BSA and d) BSA-ATPS in D_2_O buffer at *T* = 25 °C in the pressure range from 1 bar to 4.3 kbar.

The deconvoluted FT-IR spectrum of BSA is characterized by a maximum at 1651 cm^-1^, which is due to a high content of α-helical structures.^3^ The secondary structure analysis of the amide-I’ band region of BSA revealed six sub-bands. The sub-bands at 1681 cm^-1^ and 1669 cm^-1^ can be assigned to β-turn structures. The band at 1652 cm^-1^ is associated with α-helical structures, and the band at about 1637 cm^-1^ is assigned to disordered structures. The band at 1611 cm^-1^ can be associated with intermolecular β-sheets, while the band at 1628 cm^-1^ can be assigned to short-segment chains connecting α-helical segments.^3,4^

**Table S1.** Detected BSA sub-pockets and their ranks extracted from the GOLD docking runs with four different scoring metrics.

| **Sub-pocket No** | **Mean Rank** | **Rank (ASP)** | **Rank (Gold-score)** | **Rank (Chem-score)** | **Rank (Chem-PLP)** | **Pocket Residues** |
| --- | --- | --- | --- | --- | --- | --- |
| P_2_0 | 2.25 | 1 | 6 | 1 | 1 | Leu115, Pro117, Leu122, Tyr137, Ile141, Tyr160, Phe164, Ile181, Glu182, Met184, Arg185, Val188 |
| P_2_1 | 3 | 3 | 5 | 2 | 2 | Leu115, Pro117, Leu122, Glu125, Lys132, Phe133, Lys136, Tyr137, Glu140, Ile141, Tyr160 |
| P_0_1 | 3.75 | 4 | 2 | 5 | 4 | Tyr149, Glu152, Ser191, Arg194, Gln195, Arg198, Trp213, Ser214, Arg217, Leu218, Lys221, Phe222, Leu233, Leu237, Val240, His241, Arg256, Leu259, Ala260, Ile263, Ser286, His287, Ile289, Ala290, Glu291 |
| P_0_2 | 4 | 6 | 1 | 3 | 6 | Arg194, Leu197, Arg198, Ser201, Ala209, Leu210, Trp213, Arg217, Val342, Ser343, Leu346, Arg347, Glu449, Asp450, Leu452, Ser453, Leu454, Leu456, Asn457, Leu480, Val481, Asn482, Arg483, Arg484, Pro485 |
| P_2_2 | 4.25 | 2 | 8 | 4 | 3 | Pro113, Lys114, Leu115, Glu140, Ile141, Arg144, His145, Arg185, Val188, Leu189 |
| P_1_0 | 7.5 | 5 | 9 | 7 | 9 | Tyr400, Asn404, Ile407, Phe501, Phe506, Phe508, Gln521, Lys524, Gln525, Ala527, Leu528, Leu531, His534, Lys535, Val546, Met547, Phe550, Val551, Leu574, Val575, Thr578, Gln579, Leu582, Ala583 |
| P_2_3 | 8 | 7 | 14 | 6 | 5 | Phe36, Glu125, Asp129, Lys132, Phe133, Lys136 |
| P_4_1 | 8.75 | 13 | 4 | 8 | 10 | Leu189, Thr190, Ala193, Ser428, Lys431, Val432, Arg435, Tyr451, Leu454, Ile455, Arg458 |
| P_4_0 | 9.25 | 9 | 7 | 13 | 8 | Asp108, Arg144, His145, Pro146, Tyr147, Phe148, Leu189, Ser192, Ala193, Gln195, Arg196, Leu197, Ser453, Leu454, Ile455, Asn457, Arg458, Val461 |
| P_0_0 | 10.25 | 12 | 3 | 14 | 12 | Phe205, Arg208, Ala209, Lys211, Ala212, Trp213, Val215, Phe227, Val230, Thr231, Val234, Asp323, Ala324, Leu326, Gly327, Leu330, Leu346, Ala349, Lys350, Glu353, Ser479, Leu480, Val481 |
| P_1_2 | 12.5 | 15 | 13 | 15 | 7 | Glu503, Phe506, Thr507, Phe508, Phe553, Phe567, Ala568, Gly571, Pro572, Leu574, Val575 |
| P_1_3 | 15 | 8 | 29 | 10 | 13 | Leu397, Gly401, Asn404, Ala405, Val408, Glu540, Leu543, Lys544, Met547 |
| P_11 | 15.25 | 14 | 19 | 11 | 17 | Tyr155, Tyr156, Lys159, Val163, Lys180, Thr183, Met184, Lys187, His287, Glu291 |
| P_9 | 15.25 | 11 | 27 | 9 | 14 | Glu16, Glu17, Lys20, Gly21, Leu24, Phe36, Val40, Val43, Asn44, Lys131, Lys132, Trp134, Gly135, Lys136, Leu138 |
| P_2_4 | 16.25 | 10 | 25 | 12 | 18 | Pro117, Asp118, Pro119, Leu122, Tyr160, Phe164, Leu177, Leu178, Ile181 |
| P_7 | 16.75 | 19 | 18 | 19 | 11 | Arg196, Ala200, Lys204, Phe205, Asn457, Cys460, Val461, His463, Glu464, Pro467, Val472, Thr473, Cys476, Thr477, Leu480, Arg483 |
| P_14_1 | 18.5 | 21 | 17 | 20 | 16 | Lys232, Asp236, Asp258, Leu259, Tyr262 |
| P_3_0 | 19.5 | 25 | 16 | 16 | 21 | Leu386, Ile387, Asn390, Cys391, Phe394, Phe402, Leu406, Arg409, Tyr410, Lys413, Leu429, Val432, Gly433, Cys436, Cys437, Arg444, Thr448, Leu452, Leu456, Arg483, Arg484, Phe487, Ser488 |
| P_4_2 | 20.25 | 18 | 21 | 18 | 24 | Glu424, Val425, Ser428, Ile455, Arg458, Leu459, Leu462 |
| P_3_1 | 20.75 | 22 | 23 | 23 | 15 | Gln384, Ile387, Lys388, Cys391, Asp392, Glu441, Arg444, Met445 |
| P_6_0 | 21 | 16 | 24 | 22 | 22 | Thr411, Arg412, Pro415, Pro492, Asp493, Glu494, Thr495, Tyr496, Pro498, Leu532, Lys533, Pro536, Lys537 |
| P_8_0 | 22 | 20 | 12 | 30 | 26 | Val344, Arg347, Leu348, Leu379, Val380, Glu382, Pro383, Met445, Glu449, Asn482, Arg484, Pro485 |
| P_16 | 22.25 | 17 | 31 | 21 | 20 | Cys199, Ile202, Gln203, Lys242, Cys245, His246 |
| P_1_1 | 22.5 | 23 | 10 | 29 | 28 | Gln29, Tyr30, Leu31, Gln32, Gln33, Tyr84, Met87, Phe102, Leu103, His105, Lys106, Asp107 |
| P_8_1 | 22.75 | 26 | 11 | 31 | 23 | Glu382, Pro383, Leu386, Arg484, Pro485, Ser488 |
| P_5_0 | 23.5 | 30 | 22 | 17 | 25 | Leu22, Val23, Ala26, Phe27, Tyr30, Leu66, His67, Phe70, Gly71, Leu74, Glu95, Arg98, Asn99, Phe102, His246, Gly247, Asp248, Leu249, Leu250, Glu251 |
| P_14_0 | 25 | 24 | 30 | 27 | 19 | Asp236, Lys239, Val240, Asp255, Asp258, Leu259 |
| P_13_0 | 25.75 | 28 | 20 | 25 | 30 | Ser109, Ser418, Pro420, Thr421, Leu462, Lys465, Thr466 |
| P_15 | 26.75 | 31 | 15 | 34 | 27 | Ser104, Lys106, Asp107, Asp108, Tyr147, Arg196, Val461, Glu464, Lys465 |
| P_12 | 27 | 27 | 26 | 24 | 31 | Val417, Ser418, Thr421, Leu459, Leu462, His463, Thr466, Val468 |
| P_0_3 | 28 | 29 | 28 | 26 | 29 | Asp13, Leu14, His18, Leu22, Pro151, Glu152, Leu154, Tyr155, Ala253, Asp254, Arg256, Ala257, Ala260, Leu282, Leu283, Ser286 |
| P_3_2 | 33 | 35 | 34 | 28 | 35 | Pro383, Leu386, Ile387, Met445, Thr448, Glu449, Leu452, Arg484 |
| P_6_1 | 33 | 32 | 32 | 35 | 33 | Arg412, Lys413, Val414, Pro415, Gln416, Glu470, Lys471, Pro492, Asp493, Glu494, Thr495, Tyr496 |
| P_10 | 33.25 | 34 | 35 | 32 | 32 | Lys499, Ala500, Phe501, Asp502, Leu505, Phe506, Glu530, Leu531, Lys533, His534 |
| P_13_1 | 33.25 | 33 | 33 | 33 | 34 | Thr545, Val546, Asn549, Leu574, Ser577, Thr578, Ala581, Leu582 |
| P_5_1 | 36 | 36 | 36 | 36 | 36 | Ala26, Gln29, Tyr30, Leu103, Lys106, Pro146, Tyr147, Phe148, Tyr149, Ala150, Pro151, Gln195, Cys199, Cys244, Cys245, His246, Gly247, Leu249 |

**References**

1. Haiech, J. *et al.* A general framework improving teaching ligand binding to a macromolecule. *Biochim. Biophys. Acta - Mol. Cell Res.* **1843**, 2348–2355 (2014).

2. Van Holde, K. E., Johnson, W. C. & Ho, P. S. *Principles of Physical Biochemistry*. (Pearson/Prentice Hall, 2006).

3. Lu, R. *et al.* Probing the secondary structure of bovine serum albumin during heat-induced denaturation using mid-infrared fiberoptic sensors. *Analyst* **140**, 765–770 (2015).

4. Murayama, K. & Tomida, M. Heat-induced secondary structure and conformation change of bovine serum albumin investigated by Fourier transform infrared spectroscopy. *Biochemistry* **43**, 11526–11532 (2004).
